# New Trauma Score versus Kampala Trauma Score II in predicting mortality following road traffic crash: a prospective multi-center cohort study

**DOI:** 10.1186/s12873-024-01048-0

**Published:** 2024-07-29

**Authors:** John Damulira, Joshua Muhumuza, Umaru Kabuye, Godfrey Ssebaggala, Michael Lowery Wilson, Till Bärnighausen, Herman Lule

**Affiliations:** 1 Department of Surgery, Faculty of Clinical Medicine and Dentistry, Kampala International University Western Campus, Kampala, Uganda; 2 Heidelberg Institute of Global Health (HIGH), University Hospital and University of Heidelberg, Heidelberg, Germany; 3 Turku Brain Injury Centre, Department of Clinical Neurosciences, Injury Epidemiology and Prevention (IEP) Research Group, Turku University Hospital and University of Turku, Turku, Finland

**Keywords:** New Trauma score, Kampala Trauma score, Mortality, Injury outcomes, Prediction methods, Uganda

## Abstract

**Introduction:**

Mortality due to injuries disproportionately impact low income countries. Knowledge of who is at risk of poor outcomes is critical to guide resource allocation and prioritization of severely injured. Kampala Trauma Score (KTS), developed in 1996 and last modified in 2002 as KTS II, is still widely being used to predict injury outcomes in resource-limited settings with no further revisions in the past two decades, despite ongoing criticism of some of its parameters. The New Trauma Score (NTS), a recent development in 2017, has shown potential in mortality prediction, but a dearth of evidence exist regarding its performance in the African population.

**Objectives:**

To compare NTS to the modified Kampala Trauma Score (KTS II) in the prediction of 30-day mortality, and injury severity amongst patients sustaining road traffic crashes in Ugandan low-resource settings.

**Methods:**

Multi-center prospective cohort study of patients aged 15 years and above. Of the 194 participants, 85.1% were males with a mean age of 31.7 years. NTS and KTS II were determined for each participant within 30-minutes of admission and followed-up for 30 days to determine their injury outcomes. The sensitivity, specificity, and area under receiver operating characteristics curve (AUC) for predicting mortality were compared between the two trauma scores using SPSS version 22. Ethical clearance: Research and Ethics Committee of Kampala International University Western Campus (Ref No: KIU-2022-125).

**Results:**

The injury severity classifications based on NTS vs. KTS II were mild (55.7% vs. 25.8%), moderate (29.9% vs. 30.4%), and severe (14.4% vs. 43.8%). The mortality rates for each injury severity category based on NTS vs. KTS II were mild (0.9% v 0%), moderate (20.7% vs. 5.1%), and severe (50% vs. 28.2%). The AUC was 0.87 for NTS (95% CI 0.808–0.931) vs. 0.86 (95% CI 0.794–0.919) for KTS II respectively. The sensitivity of NTS vs. KTS II in predicting mortality was 92.6% (95% CI: 88.9–96.3) vs. 70.4% (95% CI: 63.0-77.8) while the specificity was 70.7% (95% CI: 64.2–77.2) vs. 78.4% (95% CI: 72.1–84.7) at cut off points of 17 for NTS and 6 for KTS II respectively.

**Conclusions:**

NTS was more sensitive but its specificity for purposes of 30-day mortality prediction was lower compared to KTS II. Thus, in low-resourced trauma environment where time constraints and pulse oximeters are of concern, KTS II remains superior to NTS.

**Supplementary Information:**

The online version contains supplementary material available at 10.1186/s12873-024-01048-0.

## Background

Annually, over 1.35 million people die from road traffic crashes (RTCs) worldwide [1]. The burden of RTC-related fatalities is 3 times higher in low-and middle-income countries (LMICs), which are anticipated to incur $834 billion dollars loss due to RTCs during 2015–2030 [2]. Uganda is one of the low-income countries that are highly burdened with mortality due to RTCs, partly due to its low-resourced prehospital care systems, limited physical, financial and human resources [3]. Over 21% of Uganda’s 49 million population live below the poverty line of US$1.90 per day, with 14% at risk of catastrophic expenditure on out-of-pocked health bills [4]. Most of the fatal RTCs result from motorcycle and car collisions, exacerbated by healthcare disparities and limited access to specialized post-injury care as more than 70% of the population reside in rural areas [5].

In addition, inadequate prevention measures such as road safety campaigns, traffic law enforcement and legislation could contribute to this burden [6]. As such, between ten to twenty people die on Ugandan roads every day from RTCs, whereas the majority of injuries result in disability [7]. The current situation necessitate improved trauma care capacity due to absence of a formal prehospital care system for immediate accident response. Efforts to establish rural trauma networks between traffic police and medical trainees who often provide the immediate accident response are still underway [8]. Sound knowledge of who is at risk of poor trauma outcomes and mortality is critical in Ugandan low-resource settings to prioritize those who are severely injured and guide the scarce resource allocation.

There is compelling evidence to suggest that trauma scores could improve outcome prediction, guide triaging and the urgency of trauma interventions, and enhance the overall quality of trauma management [9, 10]. In addition to determining prognosis, precise trauma quantification by use of scores harmonize injury communication terminology, which is critical for trauma audits, surveillance and documentation of medicolegal forensic cases [11]. A trauma score is a guide made up of parameters with codes or numerical values assigned to the ranges, whereby their summation gives an objective idea of how life threatening are the injuries sustained [12]. As such, trauma scores could be: (i) physiological which detail changes in vital signs and level of consciousness for instance the New Trauma Score (NTS) and Revised Trauma Score (RTS) that enable early clinical assessment of patients at admission; (ii) anatomical which detail the extent and number of anatomical lesions such as the Abbreviated Injury Scale (AIS) and Injury Severity Score (ISS) that allows later clinical assessment including imaging after initial patient stabilization, during surgery and autopsy or (iii) combined such as Trauma and Injury Severity Score (TRISS) and KTS which embed RTS and ISS [11]. The combined trauma scores are particularly invaluable in determining prognosis following trauma [11].

The main reason behind the development of physiological NTS and combined KTS was the feasibility for their use in resource constrained settings where initial advanced evaluation for anatomical lesions by use of computerized tomographic (CT) scans and magnetic resonance imaging (MRI) may not be available [9, 13]. The KTS was developed in 1996 [14, 15] and is the most validated and used tool in Ugandan context [13, 16–18]. However since its modification in 2002 in which the total KTS score was reduced from 16 to 10 [13], no further revisions have been made in the past two decades [19]. In the modified KTS also known as KTS II, all parameters i.e. age, number of serious anatomical injuries, systolic blood pressure, respiratory rate, and neurological status were maintained but recent studies have challenged three of its five parameters i.e. respiratory rate (RR), systolic blood pressure (SBP) ranges and Alert, Verbal Response, Pain Response, Unresponsive (AVPU) scale for its neurological assessment and called for its potential alternative score, the NTS [9, 20, 21].

Criticism for the KTS II parameters is threefold. First, it has been noted that in emergency situations with severely injured patients, RR is often manually counted for less than the recommended one minute which makes RR as a parameter subject to errors [20]. Despite automated RR machines being in existence, they are relatively expensive with limited availability in low-resource settings [22]. Therefore, peripheral oxygen saturation (SPO_2_) has been suggested as a better alternative to RR [9, 23, 24].

Secondly, KTS II begins to score a patient when SBP falls below 90 mmHg which gives the impression that all patients with SBP above 90 mmHg are normal and would have similar outcomes. On the contrary, studies show that trauma patients with SBP range of (90–109) mmHg have poorer outcomes than those with SBPs over 109 mmHg [9, 25, 26].

Lastly, although the (AVPU) parameter of KTS II is a simpler neurological assessment tool effective in the pediatric patients [27], its performance in adults has been shown to be inferior to Glasgow Coma Scale (GCS) in neurological injury severity and mortality prediction among trauma patients [21, 28, 29]. Thus, the three suggested parameters (SPO_2_, adjusted SBP ranges and GCS) constituted the development of the NTS in South Korea [9]. However, there was paucity of information on performance of NTS against KTS II in the Ugandan low resource setting despite having had good performance in a prospective study that was done in non-African setting with a sensitivity of 99% and specificity of 94% in predicting mortality among trauma patients [30]. Therefore, the primary objective in this study was to evaluate the performance of NTS versus the KTS II in prediction of mortality amongst patients involved in road traffic crashes. The secondary objectives of the study were to determine the corresponding mortality rates within each injury severity category as determined using NTS versus KTS II.

## Materials and methods

### Study design

This was a prospective multi-center cohort study involving patients presenting with road traffic-related injuries. Eligible participants were recruited consecutively.

### Study setting

This study was conducted at three tertiary and teaching hospitals in Uganda including Kampala International University Teaching Hospital (KIU-TH), Fort Portal Regional Referral Hospital (FRRH) and Hoima Regional Referral Hospital (HRRH) during November 2022-February 2023. Each of these facilities receive an average of 40–50 road traffic crash patients per month and offers 24/7 specialized emergency trauma care services [16]. The facilities were selected to represent both private (KIU-TH) and government (FRRH and HRRH) clinical practice settings in Uganda. Further, each of these facilities serve the newly created cities in Uganda which are in early phases of urban planning, including Mbarara, Fort portal, and Hoima respectively.

### Sample size determination and sampling

Sample size was calculated using Daniel’s formula (1999) [31].

$n = {z^2}p(1 - p) \div {d^2}$ ; where n was minimum sample size required, z was standard normal deviate (1.96 at 95% confidence interval), *p* was the proportion of patients who sustained road traffic crash-related injuries from the region of South-western Uganda where the current study was conducted, relative to the entire country i.e., (13.3%) based on Muni et al. [7], d was the accuracy level (margin error) of 0.05. Substituting into the formula,

$$\begin{array}{l}\:n = \left( {1.96\: \times \:1.96} \right) \times \:0.132\:\left( {0.868} \right)\\\div 0.0025 = 176\,patients\end{array}$$

Adding 10% of anticipated loss to follow-up resulted in a final sample size of 194 patients.

### Eligibility criteria

To be eligible, it was a requirement that participants sustained road traffic crash-related injuries, be presented to any of the study sites within 24 h after injury and were alive at the time of admission. Since NTS had not been validated in patients under 15 years, we limited the study to patients aged 15 years or more. We excluded patients who: presented after 24 h, came in as referrals from other hospitals after initiation of trauma resuscitation, confirmed dead at the time of arrival, and those who sustained trauma outside of road traffic environment. All parameters of the scores were obtained within the first 30 min of doctor-patient interaction at admission to capture vital signs required to guide immediate trauma resuscitation during primary survey assessment. The parameters were later summed up and transferred from case files to the questionnaire after secondary survey and patient stabilization. This approach intended to depict the typical workflow and applicability of the tools by the end user (clinicians). Each participant was recruited once using a unique code to avoid duplication during follow-up that could arise from inter-hospital transfers.

### Data collection procedure and quality control

Data was collected by JD with the help of research assistants (RAs). RAs were doctors allocated to accident and emergency (A & E) units of KIU-TH, FRRH and HRRH. The RAs were trained in two sessions (one at the beginning of the data collection process and a refresher midway) on how to assess patients using both trauma scores. The data collection tool was designed in English and translated in commonly used local languages (Runyankore and Runyoro-Rutooro) to enhance effectiveness in communication. For validity, the questionnaire was pretested and appropriated before the data collection process and a content validity index of 0.8 was considered appropriate. Data completeness was ensured for each entry prior closing patients’ case files and through reminder phone calls for follow-up appointments.

### Study variables

Each participant was scored using both NTS and KTS II. Blood pressure was measured using the same type of digital sphygmomanometers (Heartipro^®^) placed on upper arm and the same type of FDA approved pulse oximeters (Oxiline Pulse 7 S Pro) were used on the index fingers. Data including patients’ sociodemographic, road user category, mechanism of injury, time to hospital, comorbidity, chronic medication use, physical examination findings and follow up details were collected using a questionnaire that was specifically designed for this study (supplementary material 1).

NTS and KTS II were the independent variables. Mortality within 30 days was the dependent variable. The 30-day duration of follow-up was chosen because studies which used 14 days had cited short duration of follow up as a limitation [32]. The mortality data was collected by RAs by retrieval of all case files from accident and emergency department, surgery wards, theatre logbooks, and through cross-validation with mortuary records regarding death certification at the respective hospitals.

Regarding NTS, peripheral oxygen saturation, systolic blood pressure and Glasgow coma scale are its parameters and the injury severity categories were defined as [18–23] for mild, [12–17] moderate, and [3–11] severe [9]; whereas for KTS II, respiratory rate, systolic blood pressure, AVPU scale, age and number of serious injuries are its parameters. The injury severity categories were (9–10) for mild, (7–8) moderate, and (≤ 6) severe as classified in previous studies [33]. Injury outcome within 30-days of follow-up was dichotomized as survived or died.

### Statistical analysis

Data were entered into Microsoft Excel 2010 and thereafter exported to SPSS version 22 for Windows for analysis. Descriptive statistics were used to analyze the baseline participant characteristics.

The primary objective was obtained by using Receiver Operator Characteristic (ROC) curve analysis. Appropriate cut off points were determined using ROC for both NTS and KTS II and used to determine their sensitivity and specificity in predicting mortality among RTC patients. The area under the ROC curve (AUC) for the two scores was compared.

To achieve the secondary objectives, the number of patients in each injury severity group was expressed as a percentage of the total RTC patients, and the number of mortalities in each severity category was expressed as a percentage of the total number of patients in that severity category.

### Ethical considerations and consent

The research and all methods were performed in accordance with the ethical standards stipulated in the declaration of Helsinki and its later amendments, and in accordance with relevant local guidelines and regulations. Ethical approval was obtained from the Research and Ethics Committee of Kampala International University Western Campus (**Ref No: KIU-2022-125**). Written informed consent was obtained from each participant or their legally authorized representatives as appropriate before recruitment into the study.

## Results

### Baseline characteristics of the study participants

The baseline characteristics of study participants are summarised in (Table 1). By the end of the study period, 230 trauma patients were seen at the study sites, 29 of them were not eligible and 7 declined to participate. This left 194 patients who were eligible and consented to participate of which 27 (13.9%) died by the end of 30-days follow-up. The majority were males 165 (85.1%), aged 25–39 years 98 (50.5%), and presented within 30 min of injury 168 (86.6%), with a mean pre-hospital time of 20 min (SD = 11.5). Mostly, the participants were pedestrians and motorcyclists who had sustained a variety of injuries affecting the head, limbs, chest, and abdomen.

**Table 1:** Baseline characteristics of study participants

Characteristic	Frequency	Percentage	95% CI
Age category			
15–24	58	29.9	23.2–36.6
25–39	98	50.5	43.8–58.2
40–59	30	15.5	10.8–20.6
>=60	8	4.1	1.5–7.2
Sex			
Male	165	85.1	79.9–89.7
Female	29	14.9	10.3–20.1
Residence			
Rural	64	33.0	26.3–39.7
Urban	130	67.0	60.3–73.7
Other	20	10.3	5.7–14.4
Time to presentation (min) Mean = 20.0, SD = 11.5, Min = 5.0, Max = 60.0			
Within 30 min	168	86.6	82.0-91.2
Over 30 min	26	13.4	8.8–18.0
Road user			
Pedestrian	74	38.1	31.4–44.8
Cyclist	48	24.7	18.0-30.4
Driver	16	8.2	4.6–12.4
Motorcycle passenger	56	28.9	22.7–35.6
Cause of accident			
Driving/ riding errors	115	59.3	51.5–66.0
Drug/ alcohol abuse	37	19.1	13.9–24.7
Others	42	21.6	16.0-27.8
Chronic medication			
None	148	76.3	70.6–82.0
Anticoagulants	1	0.5	0.0-1.5
Steroids	2	1.0	0.0-2.6
NSAIDs	5	2.6	0.5–5.2
Other	38	19.6	13.9–25.3
Comorbidity			
None	148	76.3	70.6–82.0
DM	9	4.6	1.5–7.7
HTN	11	5.7	2.6–9.3
DM + HTN	6	3.1	1.0-5.7
HIV	11	5.7	2.6–9.3
Other	9	4.6	2.1–7.7
Head injury			
No	60	30.9	24.7–37.6
Yes	134	69.1	62.4–75.3
Chest Injury			
No	140	72.2	66.0-78.4
Yes	54	27.8	21.6–35.0
Abdomen and pelvis			
No	160	82.5	77.3–87.6
Yes	34	17.5	12.4–22.7
Limb Injuries			
No	136	70.1	63.4–76.8
Yes	58	29.9	23.2–36.6
Hospital			
HRRH	80	41.2	34.5–48.5
FRRH	82	42.3	35.1–49.5
KIU-TH	32	16.5	11.3–22.2

*DM = Diabetes mellitus*,* HTN = Hypertension*,* HIV = Human immune deficiency virus*,* HRRH = Hoima regional referral hospital*,* FRRH = Fort Portal regional referral hospital*,* KIU-TH = Kampala international university teaching hospital*,* SD = Standard deviation*,* Min = Minimum*,* Max = Maximum*,* CI = Confidence interval.*

### Comparison of NTS to KTS II in the prediction of mortality among RTC patients

In this study, the area under curve for NTS was higher (0.869, *p* < 0.001) than that for KTS II (0.857, *p* < 0.001). The NTS predicted mortality with a sensitivity of 92.6% and specificity of 70.7% at cut off 17 whereas the KTS II predicted mortality with a sensitivity of 70.4% and specificity of 78.4% at a cut off 6 as shown in Table 2.

**Table 2:** NTS versus modified KTS II in prediction of mortality

Score	Sensitivity	Specificity	AUC	*p* value
NTS at 17 (95%CI)	92.6% (88.9–96.3)	70.7% (64.2–77.2)	0.869 (0.808–0.931)	< 0.001
KTS II at 6 (95%CI)	70.4% (63.0-77.8)	78.4% (72.1–84.7)	0.857 (0.794–0.919)	< 0.001

*NTS = New trauma score*,* KTS II = Modified Kampala trauma score*,* AUC = Area under the curve*,* CI = Confidence interval*,* P value = Probability value*

### Injury severity among RTC patients as classified by NTS versus KTS II

**Fig. 1:**
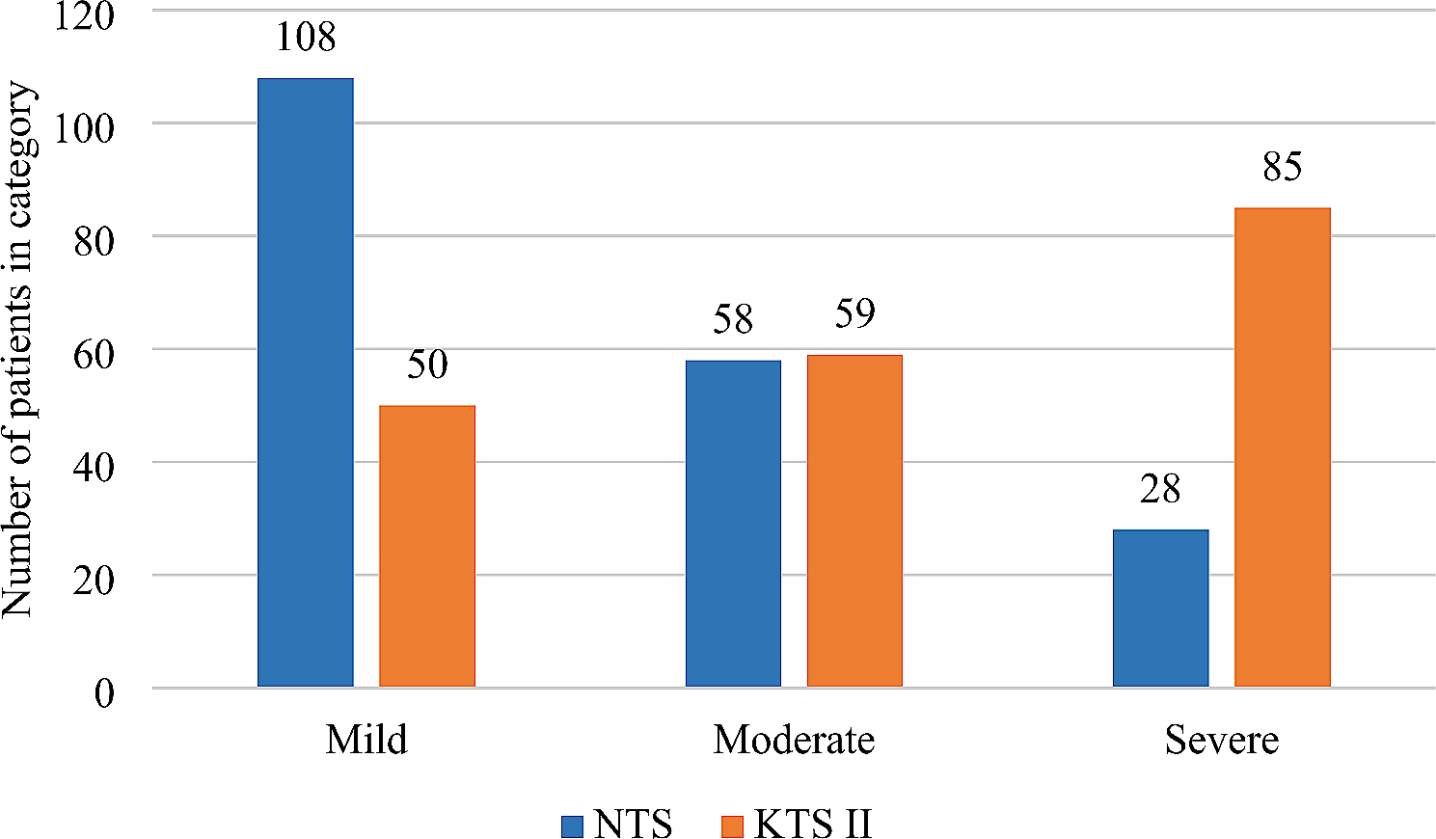
Injury severity among RTC patients as classified by NTS versus KTS II

As shown in Fig. 1, NTS categorized most patients as mild 108 (55.7%) whereas KTS II had the majority in the severe category 85 (43.8%). Both scores nearly agreed for their moderate categorization at 58 (29.9%) and 59 (30.4%) for NTS and KTS II respectively.

### Mortality rates within each injury severity group as classified by NTS versus KTS II

As illustrated in Table 3, the mortality rates for the various injury severity categories based on NTS were 50.0% (severe), 20.7% (moderate), and 0.9% (mild) but when assessed using KTS II, the respective mortality rates were 28.2%, 5.1% and 0% for the severe, moderate, and mild injury categories. Both scores showed a statistically significant association in which mortality increased with worsening injury severity (*p* < 0.001).

**Table 3:** Mortality rates in each injury severity group as classified by NTS versus KTS II

Severity category	NTS *N*(%)	KTS II *N*(%)	*P* Value
Mild	1(0.9%)	0(0.0%)	< 0.001
Moderate	12(20.7%)	3(5.1%)	
Severe	14(50.0%)	24(28.2%)	

KTS II = Modified Kampala trauma score, NTS = new trauma score

## Discussion

This study aimed at comparing the new trauma score (NTS) to the modified Kampala Trauma Score (KTS II) in the prediction of 30-day mortality, and injury severity amongst patients sustaining road traffic crashes. We found that the sensitivity of NTS in mortality prediction was higher than KTS II (92.6% vs. 70.4%). We did not find literature directly comparing NTS to KTS II. However, when we evaluated studies that have reported either on NTS or KTS II, the sensitivity of NTS in our study was slightly lower than 99% reported in a prospective Iranian study of 544 participants at level III trauma centre in which NTS was compared to the revised trauma score (RTS) and Glasgow Age Pressure (GAP) score, whose sensitivities for mortality prediction were 98% and 97% respectively [30]. The discrepancy could arise from exclusion of patients with single injuries and those younger than 18 years in the Iranian cohort [30].

Further, our findings showed that KTS II was more specific in mortality prediction than NTS, with specificity of (78.4% vs. 70.7%) respectively. The specificity of KTS II in the present study is close to 81% reported in a previous prospective single centre study of 173 RTC patients in South-western Uganda where KTS II was reported superior to the new injury severity score (NISS) that had 78.4% [34]. However, unlike the present study, Mutooro et al. [34] only included patients aged 18 years and above with multiple trauma of 3 or more injuries, and as such, their findings may not be applicable to those with isolated injuries.

In addition, we found that both KTS II and NTS had comparable performance in terms of AUC i.e. (0.86 vs. 0.87) respectively (*p* < 0.001). The AUC performance of KTS II in our study is comparable to 0.87 reported in Western Uganda when it was being compared to NISS [34] but for NTS, our AUC was lower than 0.919 reported from South Korea where NTS was first developed [9]. In the prospective Korean validation study of 3106 trauma patients aged 15 years and above, NTS was shown to have a better performance in mortality prediction when compared to GAP and RTS with AUC of 0.912 and 0.906 respectively [9].

In our comparative analysis of injury severity classification based on NTS vs. KTS II as the secondary objective, we found that NTS categorized 55.7% of patients as mild, 29.9% moderate and 14.4% severe, thus the proportions in each category decreased with increasing injury severity. This agreed with the findings from two retrospective studies that have used NTS of which one was conducted using data from two tertiary hospitals in Iran on multiple trauma patients aged 18 years and above where the proportions of mild, moderate, severe were 99%, 0.7% and 0.3% respectively [35]. The other study was conducted using data from a level I trauma centre in France where 1001 trauma patients were included regardless of their age and number of injuries [36]. In French study [36], NTS classified 81% of participants as mild, 9.7% moderate and 9.3% severe.

On the other hand, in the present study KTS II categorized 25.8% of patients as mild, 30.4% moderate and 43.8% severe, therefore the number of patients increased with severity. This was contrary to the findings from a retrospective study of 362 trauma patients that was conducted using data from a tertiary hospital in Ethiopia where KTS II classified 82.3% of their participants as mild, followed by moderate 11.1% moderate and 6.6% severe [37]. However, the Ethiopian study included participants irrespective of age and documented excluding incomplete records.

In this study, both NTS and KTS II agreeably classified approximately 30% of all RTC patients as being moderately injured. They however varied in their categorization of mildly and severely injured patients whereby for NTS majority were in the mild category whereas for KTS II, majority were in the severe category. The differences could be explained by the fact that NTS is solely made up of physiological parameters while KTS II additionally constitutes an anatomical parameter regarding number of serious injuries and age [9, 34].

Furthermore, the overall mortality rate among RTC patients in this study was 13.9% which is slightly lower than the 14.45% reported from a single-center study that was previously conducted at a university teaching hospital in Western Uganda [34], possibly because the latter only included multiply injured patients with three or more injuries. In terms of proportions of fatalities within each injury severity category, NTS predicted higher mortality rates than KTS II across all the injury severity groups i.e., (0.9% vs. 0%) mild, (20.7% vs. 5.1%) moderate and (50% vs. 28.2%) severe, respectively. Scientists in France documented similar findings where NTS predicted higher mortality rates when its being compared to MGAP score which combines mechanism of injury (M), Glasgow coma scale (G), patients’ age (A) and systolic blood pressure (P) hence the acronym (MGAP); thus when assessed using NTS vs. MGAP, their predicted mortality rates for each injury severity category were (22% vs. 1%) in the mild, (42% vs. 12%) in moderate group and (100% vs. 43%) amongst the severely injured group respectively [36].

Our findings could point to the high sensitivity and low specificity of NTS against KTS. For instance, in the present study, out of the 108 patients classified as mild by NTS, 1 (0.9%) died whereas out of the 50 patients classified as mild by KTS II, none (0%) died. This potentially misguiding categorization by NTS was also noted in its moderate category where 12 out of 58 (20.7%) of patients died compared to only 3 out of 59 (5.1%) patients in KTS II moderate category. Our findings could be correlated to the study in France where 1.7% (*n* = 14) out of 810 trauma patients who were classified in the mild injury category based on NTS subsequently died within 30 days [36]. This could result in serious consequences in LMICs where less severely injured patients are discharged early before 30 days due to high bed occupancy and overcrowding in hospitals. The ability of a score to categorize those likely to die as severely injured is a critical step in the triaging process and informing scarce resource allocation in LMICs.

Lastly for the severe category, mortality rates were higher based on NTS 50% (14/28) vs. KTS II 28.2% (24/85). In conformity with the present study, other scholars have reported higher mortality rates in the severe injury subgroup both based on NTS: 70% (7/10) [35], and KTS II: 45.7% (16/35) [34], with NTS having higher proportions.

### Study strengths and limitations

To the best of the authors’ knowledge, this is the first study to compare NTS and KTS II. Being a prospective cohort study with specifically designed data collection tools, the data quality through follow-up for completeness of the data items were achieved. However, this study was not without limitations. First, our relatively smaller sample and consecutive recruitment could limit the generalizability of our findings. Secondly, inter-rater variation among the research assistants could have existed despite training sessions on the use of both trauma scores. Thirdly, the ease of implementation of NTS versus KTS II by the research assistants was not assessed, which may be construed as a drawback since trauma scores should become part of the documentation of a patient after assessment. Fourth, 30-day follow-up is considered short term, thus mortalities beyond 30 days could have been missed. Lastly, we prioritized road traffic crashes, being the huge trauma burden in our settings but comparing the performance of NTS to KTS II in other trauma population such as assaults, fall from heights and burns would yield more representative results.

## Conclusion

NTS was more sensitive but less specific compared to KTS II in predicting 30-day mortality following road traffic crash across all injury severity categories. Thus, in a Ugandan low resource trauma environment where time constraints and pulse oximeters are of concern, KTS II remains superior to NTS. These findings could be applicable to triaging teams in low-resource settings. Future studies should be long-term follow-up cohorts to compare the two tools in a diverse trauma population including those under 15 years of age, putting into consideration the performance of both in predicting morbidity outcomes.

### Electronic supplementary material

Below is the link to the electronic supplementary material.

Supplementary Material 1

## Data Availability

No datasets were generated or analysed during the current study.

## Declarations

### Guarantors

JD, HL.

## Abbreviations

NTS: New trauma score
KTS: Kampala trauma score
KTS II: Modified Kampala trauma score
RTC: Road traffic crash
KIU-TH: Kampala international university teaching hospital
FRRH: Fort portal Regional Referral Hospital
HRRH: Hoima Regional Referral Hospital
LMICs: Low-and Middle-income countries
