# Supplementary material for: New Trauma Score versus Kampala Trauma Score II in predicting mortality following road traffic crash: a prospective multi-center cohort study
Source: BMC Emerg Med. 2024 Jul 29;24:130. doi: 10.1186/s12873-024-01048-0 (PMC11287828; doi:10.1186/s12873-024-01048-0)
Supplement: Supplementary file 1 — Supplementary Material 1 [file 12873_2024_1048_MOESM1_ESM.pdf]

Appendix VA: Data collection tool. (English version)

Introduction

My name is Dr Damulira John, a post graduate (master's) student at Kampala international University (KIU). Am the principle investigator in this research study aimed at evaluating New trauma score versus Kampala trauma score II at predicting mortality among road traffic accident patients. The study includes a follow up period of thirty days from today during which you will receive once weekly checks from us to check on how you are improving. If discharged, the follow up checks will be continued in form of phone calls to finish the thirty days.

Protecting Data Confidentiality

This information which you are going to give us will be kept confidential. Your name will not be included on this form to make sure that your identity remains unknown. The hard copies shall be kept in locked shelves, and will be destroyed at a set time after completing this research. Soft copies will be saved in password protected files.

Right to Refuse/Withdraw

Participation into this research study is at free will. You reserve the right to refuse to join and if after joining, you decide to leave at any time. I thank you for choosing to join.

<table>
<tr><td colspan="8">Patient demographics</td></tr>
<tr><td>Initials of patient's name</td><td></td><td>Age (only 15years plus, are eligible)</td><td></td><td colspan="2">Patient Identification Number (PIN)</td><td colspan="2"></td></tr>
<tr><td>Patient's phone number(s).</td><td></td><td>Sex</td><td>M</td><td>F</td><td>Education</td><td colspan="2"></td></tr>
<tr><td>Occupation</td><td></td><td>Marital status</td><td></td><td>Religion</td><td colspan="3"></td></tr>
<tr><td>Village</td><td></td><td>Parish</td><td></td><td>Subcounty</td><td></td><td>District</td><td></td></tr>
<tr><td>Tribe</td><td></td><td colspan="3">Next of kin's phone number(s)</td><td colspan="3"></td></tr>
<tr><td colspan="8">History</td></tr>
</table>

KIU RESEARCH ETHICS COMMITTEE(REC)
APPROVED
VALID UNTIL
KIU
09 NOV 2023
P.O. Box 71, Bushenyi - Ishaka

Date of admission			Time of admission		
Road User Category (Tick as appropriate)					
Pedestrian	Cyclist (motorcycle or bicycle)	Passenger	Driver	Other (Specify)	
Time of accident (RTA)					
Likely Cause Of Accident (Tick as appropriate)					
Alcohol abuse or influence of other drugs	Over speeding	Other cause (specify)			
Long term medication use (Tick as appropriate)					
Anticoagulants	Steroids	NSAIDS	Other (Specify)		
Comorbidities (Tick as appropriate)					
DM	HTN	HIV	COPD	Other (specify)	

**Examination findings**						
Respiratory rate (RR)		Systolic blood pressure (SBP)				
Peripheral oxygen saturation (SPO_2)		Glasgow Coma Scale (GCS)				
Alert, Verbal, Pain, Unresponsive (AVPU) scale		Number of serious Injuries				
Body region injured (Tick as appropriate)						
Head	Neck	Chest	Abdomen	Pelvis	Upper Limbs	Lower limbs
**Total score**						

KIU RESEARCH ETHICS COMMITTEE(REC)
APPROVED
VALID UNTIL
09 NOV 2023
P.O. Box 71, Bushenyi - Ishaka

**NTS**			**KTS II**		
**Initial Resuscitation at A&E** (tick as appropriate).					
Airway	Breathing		Circulation	Other (specify)	
**Diagnosis of the patient's condition**					
**Disposition** (destination from A&E)					
Home			Declinedadmission		
Referred			Reason for referral		
Admitted	Surgical ward		Theatre	ICU	
**Follow up**					
	Week one	Week two	Week three	Day 30	
Convenient day & time to patient					
If alive, write complaint(s) &action(s) taken					
If otherwise, write date of death					

KIU RESEARCH ETHICS COMMITTEE(REC)
APPROVED
VALID UNTIL
09 NOV 2023
KIU
P.O. Box 71, Bushenyi - Ishaka
